# Supercooling Pretreatment Improves the Shelf-Life of Freeze-Dried *Leuconostoc mesenteroides* WiKim32

**DOI:** 10.4014/jmb.2209.09022

**Published:** 2022-10-26

**Authors:** Seul-Gi Jeong, In Seong Choi, Ho Myeong Kim, Ji Yoon Chang, Hae Woong Park

**Affiliations:** 1 Technology Innovation Research Division, World Institute of Kimchi, Gwangju 61755, Republic of Korea; 2 Public CMO for Microbial-based Vaccine, Hwasun-gun, Jeollanam-do 58141, Republic of Korea

**Keywords:** Lactic acid bacteria, *Leuconostoc mesenteroides*, supercooling pretreatment, exopolysaccharide, storage stability

## Abstract

Storage stability of freeze-dried lactic acid bacteria is a critical factor for their cost-effectiveness. Long-term storage of lactic acid bacteria enables microbial industry to reduce distribution costs. Herein, we investigated the effect of cold adaptation under supercooling conditions at −5°C on the viability of *Leuconostoc mesenteroides* WiKim32 during the freeze-drying process and subsequent storage. Cold adaptation increased the thickness of exopolysaccharides (EPS) and improved the viability of freeze-dried *Leu. mesenteroides* WiKim32. Compared to non-adapted cells, cold-adapted cells showed a 35.4% increase in EPS thickness under supercooling conditions. The viability of EPS-hydrolyzed cells was lower than that of untreated cells, implying that EPS plays a role in protection during the freeze-drying process. Cold adaptation increased the storage stability of freeze-dried *Leu. mesenteroides* WiKim32. Fifty-six days after storage, the highest viability (71.3%) was achieved with cold adaptation at −5°C. When EPS-containing broth was added prior to the freeze-drying process, the viability further increased to 82.7%. These results imply that cold adaptation by supercooling pretreatment would be a good strategy for the long-term storage of *Leu. mesenteroides* WiKim32.

## Introduction

Lactic acid bacteria (LAB) are Generally Recognized as Safe (GRAS) organisms successfully used in the fermented food industry, such as fermented meat products, fermented vegetables, and dairy products [1]. LAB species are added during the fermentation process to improve the quality of fermented foods [2, 3]. During fermentation, LAB plays a critical role in the improvement of sensory characteristics, shelf-life extension, and promotion of functionality of foods [4]. As quality control is an important factor for the commercialization of various fermented foods, the demand for LAB starters has increased; therefore, mass production of LAB starters is required to ensure cost-effectiveness.

Commercialized LAB starters should be highly viable during the formulation and highly stable during subsequent storage. Freezing and freeze-drying methods are commonly used to maximize the cell viability and storage stability of LAB starters. However, these methods cause LAB starters to face unfavorable conditions such as ice crystal formation and osmolarity stress during the process, resulting in cell membrane damage [5].

Various cryoprotectants are introduced to limit the negative effects caused by low-temperature conditions. These agents are mixed with LAB starters before the freeze-drying process to ensure viability [6]. Depending on their molecular weight, these agents are categorized into two types: low-molecular-weight agents and high-molecular-weight agents. The former ones, *i.e.*, lactose, sucrose, and trehalose, prevent denaturation and damage caused by low water content, forming hydrogen bonds with polar groups and proteins of the cell membrane [7]. The latter ones, *i.e.*, skim milk, maltodextrin, and soybean powder, are primarily used in LAB protection due to their non-toxicity, availability, and biodegradability [8, 9].

LAB can develop freezing tolerance in response to physiological stress, *i.e.*, exposure to cold conditions before the freeze-drying process. Temperatures below the optimum temperature required for LAB growth result in the initiation of cold shock responses that improve LAB viability. These responses include upregulation of cold-related genes, production of cold-induced proteins, changes in fatty acid composition, and an increase in cation influx [10-13]. Cold-adapted cells eventually survive after the freeze-drying process. Although the abovementioned studies have focused on *Lactobacillus* spp., the long-term storage of other genera of LAB after freeze-drying has recently attracted increasing attention.

Sensory characteristics of fermented vegetable foods, such as kimchi, pao cai, and sauerkraut, can be enhanced using *Leuconostoc* spp. *Leu. mesenteroides* WiKim32, isolated from kimchi at an early stage of fermentation, has been used as a kimchi starter owing to its advantageous traits; particularly, it extends the shelf-life and improves the sensory properties of kimchi [14]. Our previous study revealed that supercooling pretreatment at −5°C improves the storage stability of *Lac. brevis* WiKim0069 [13]. The overall objective of this study was to examine the effects of cold adaptation on exopolysaccharides (EPS) production and cell wall composition, which improve the viability of *Leu. mesenteroides* WiKim32. Furthermore, the effect of adding various concentrations of EPS before the freeze-drying process on cell viability was investigated.

## Materials and Methods

### Bacterial strain and Culture Conditions

*Leu. mesenteroides* WiKim32 (KFCC11639P), isolated from kimchi and developed as a kimchi starter, was obtained from the culture collection at the World Institute of Kimchi (Gwangju, Republic of Korea). Stock cultures were stored at −80°C (MDF4V; Panasonic, Japan) in De Man, Rogosa, and Sharpe (MRS) medium (Difco Laboratories, USA) with 20% glycerol. Working cultures were grown in MRS medium at 30°C for 24 h.

### Cold Adaption Procedure and Cell Viability

The cold adaptation process and measurement of cell viability were carried out using the procedure described by Choi *et al*. [13]. *Leu. mesenteroides* WiKim32 was grown in MRS medium at 30°C for 24 h and harvested by centrifugation (10,000 ×*g* at 25°C for 20 min). The extra MRS broth was stored at 4°C as a suspension solution for subsequent experiments. The precipitated cells collected by centrifugation were washed twice with sterile phosphate-buffered saline (3M Company, USA) and concentrated to 4 × 10^10^ CFU/ml in either saline or the 24 h-cultured MRS broth. The final cell concentration was adjusted to 2 × 10^10^ CFU/ml with a uniform volume of sterile 20% solution of soy powder as cryoprotectant. The cells suffered from cold stress for 2 h at 10°C or −5°C prior to freeze-drying. A control sample was not exposed to cold stress. The freeze-drying process was conducted at −80°C with a vacuum degree lower than 1 Pa using a freeze dryer (FDCF-12012; Operon, Republic of Korea). The freeze-dried cells were separately prepared for each storage period and kept for 56 days at −20°C.

Cell viability was evaluated using the plate count method instantly after freeze-drying and during storage periods. The appropriate diluent was poured onto MRS agar and incubated at 30°C for 48 h. Bacterial colonies were counted and calculated as CFU/ml.

### Preparation of EPS and EPS-Free Bacteria

**EPS purification.** The EPS was purified following the procedure reported by Choi *et al*. [15], with minor adjustments. After cell harvesting, discarded MRS broth was boiled at 100°C for 5 min for inactivated enzymes and the supernatants were obtained by centrifugation (10,000 ×*g* for 20 min at 4°C). For ethanol precipitation and lyophilization, three times the amount of 95% ethanol was added to the supernatants and kept at −20°C overnight before centrifugation (10,000 ×*g* for 20 min at 4°C). The protein was removed by dissolving it in distilled water and deproteinizing it with 4% trichloroacetic acid (Sigma-Aldrich, USA). Crude EPS was reprecipitated with 95%ethanol, dissolved in distilled water, dialyzed with distilled water for 2 days at 4°C using Slide-A-Lyzer dialysis cassettes (10K MWCO; Thermo Scientific, USA), and dried at −20°C overnight. Purified EPS was added to the cell viability test as a cryoprotectant.

**Enzymatic hydrolysis of EPS**. To reduce the EPScontent of *Leu. mesenteroides* Wikim32, bacterial cells were treated with hydrolysis enzyme, Dextranase Plus L (Novozymes, *Chaetomium erraticum*). Subsequently, 500 μl of Dextranase solution was added to 200 ml of bacterial culture broth. Enzymatic hydrolysis was performed for 12 h at 30°C. The EPS-hydrolyzed cells were harvested by centrifugation at 10,000 ×*g* for 20 min at 4°C and washed with distilled water three times. The viability of EPS-hydrolyzed cells was measured with or without 0.1% purified EPS as a cryoprotectant using the plate count method.

### Monosugar Composition

Monosaccharide compositions were analyzed following the method described by Choi *et al*. [15]. The neutral sugars of cold-adapted and non-adapted cells was acidic hydrolyzed, converted into their corresponding alditol acetates, and measured using a gas chromatography apparatus (7890A; Agilent Technology, USA) equipped with a flame ionization detector. A capillary column (DB-225; 30 m length, 0.25 mm diameter, 0.25 μm film thickness; Agilent Technology), wherein the temperature was controlled at 100–220°C with an increasing rate of 5°C/min, was used.

### Transmission Electron Microscopy (TEM)

The morphological changes of cold-adapted cells were analyzed using a transmission electron microscope (JEM-1400; Jeol, Japan). Prior to observation, each sample fixed in 2% glutaraldehyde (Merck, Germany) was post-fixed overnight in 1% osmium tetroxide (Sigma-Aldrich), dehydrated with a graded ethanol series of 30%, 50%, and 70%, embedded in LR white acrylic resin (Sigma-Aldrich) at 50°C for 24 h, and then sectioned using an ultra-microtome with a diamond knife. The sections were placed on the center of a TEM grid and stained with uranyl acetate (Sigma-Aldrich) and lead citrate (Sigma-Aldrich). EPS thickness was examined using DigitalMicrograph software (Gatan Inc., USA). The average diameter was calculated from the values for 16 bacteria taken at random locations of each cell.

### Statistical Analysis

All experiments were carried out three independent experiments. All data were analyzed by analysis of variance using Statistical Package for the Social Sciences software (version 19.0; IBM Corp., USA). Mean values were analyzed using Duncan’s multiple tests to determine significant differences in the treatments at *p* < 0.05.

## Results and Discussion

### Effect of cold adaption on EPS thickness of *Leu. mesenteroides* WiKim32

**Fig. 1:**
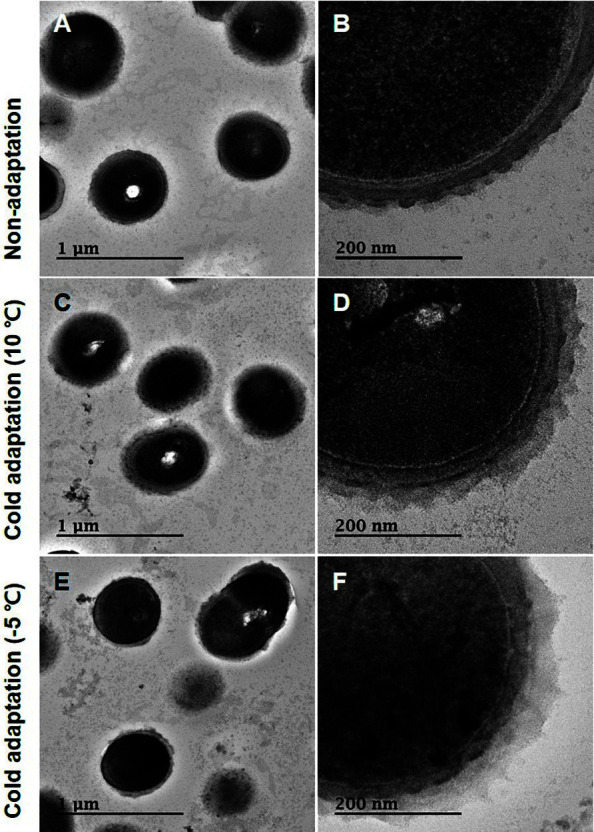
TEM image of *Leuconostoc mesenteroides* WiKim32. Control (non-adapted) cells (**A, B**) and cells cold-adapted at 10°C (**C, D**) or −5°C (**E, F**). Exopolysaccharide is attached to the cell wall (black arrow). Scale bars indicate 1 μm and 200 nm.

Structural changes in bacterial cell walls were determined using transmission electron microscopy (TEM)(Fig. 1). EPS thickness of *Leu. mesenteroides* WiKim32 varied with pretreatment conditions (*F* = 62.4, *df* = 2,45, *p* < 0.001). The thickest EPS (51.6 ± 3.52 nm) was observed in cold-adapted cells at −5°C, followed by those in cold-adapted cells at 10°C (42.0 ± 3.34 nm; Table 1). Compared with that of non-adapted cells, EPS thickness of cold-adapted cells increased by 10.2–35.4%. Monosugar composition strongly supported the reason EPS thickness depended on adapted conditions. Carbohydrates in non-adapted cells are mainly composed of arabinose (1.6%), galactose (2.8%), and glucose (8.7%) (Table 2). After cells were adapted at −5°C, carbohydrate contents were changed to arabinose (1.7%), galactose (2.9%), and glucose (9.6%). There are four structural models for cell walls of LAB: (a) cell wall composed of the outer layer, peptidoglycan, and lipoteichoic acids; (b) cell wall enveloped by EPS; (c) cell wall surrounded by the surface layer protein (SLP); (d) cell wall crosslinked EPS and SLP [13]. Among these models, *Leu. mesenteroides* WiKim32 has a cell wall model in which EPS is attached to the cell wall [15]. In this study, supercooling pretreatment further increased the EPS thickness of *Leu. mesenteroides* WiKim32.

### Effect of Enzymatic Hydrolysis of EPS on Cell Viability

**Fig. 2:**
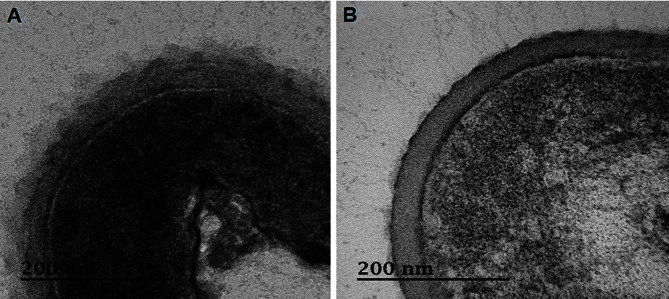
TEM image of *Leuconostoc mesenteroides* WiKim32 as control cells (untreated cells) (A) and enzymatically hydrolyzed cells (EPS removed cells) (B).

**Fig. 3:**
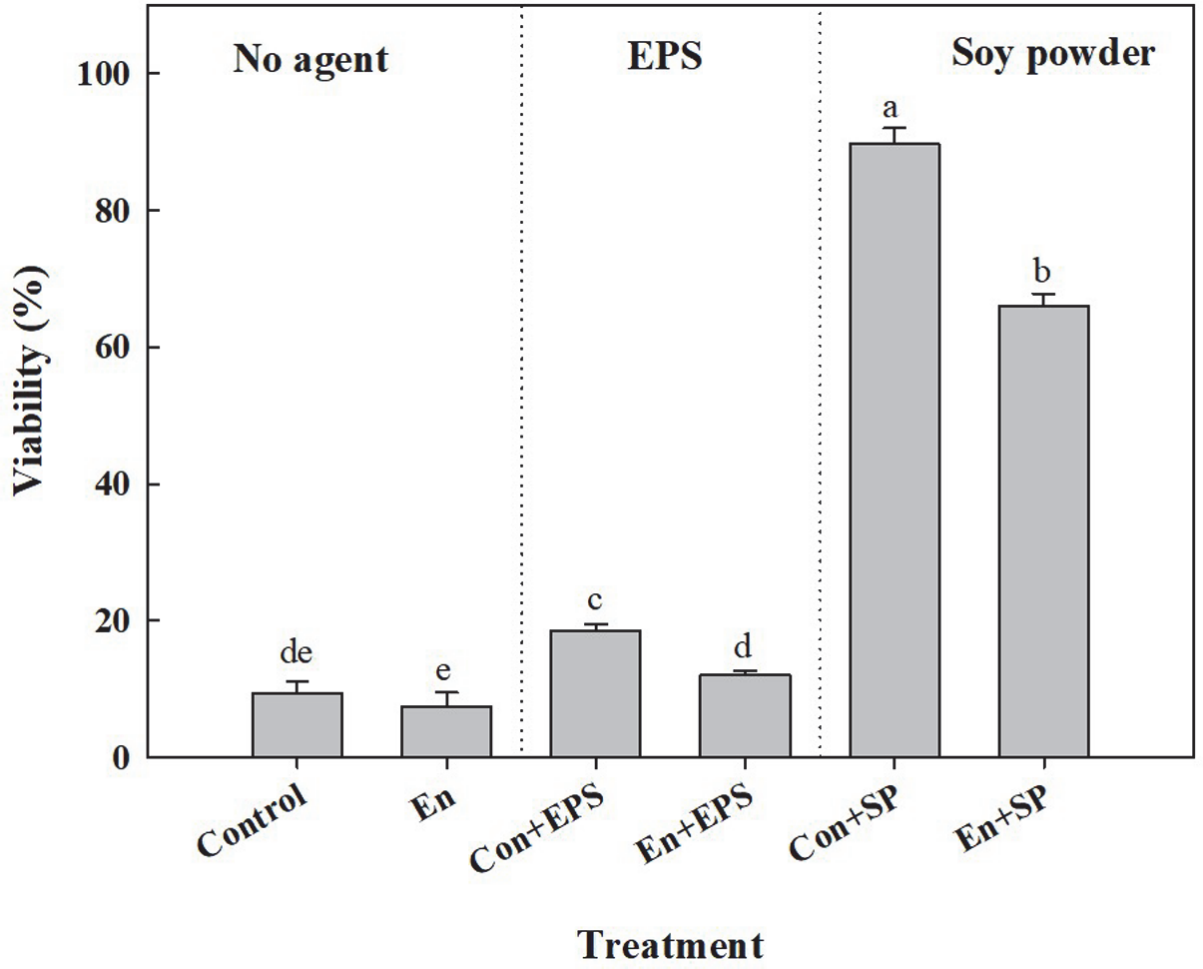
Viability of *Leuconostoc mesenteroides* WiKim32 depending on the presence of EPS and cryoprotective agent. Control: untreated cells; En: enzymatically hydrolyzed cells; Con+EPS: free cells + EPS; En+EPS: enzymatically hydrolyzed cells + EPS; Con+SP: free cells + soy powder; En+SP: enzymatically hydrolyzed cells + soy powder.

To understand the role of EPS in the viability of *Leu. mesenteroides* WiKim32, EPS-hydrolyzed cells were manufactured (Fig. 2). The glucose content was reduced by hydrolysis of EPS attached to the cell wall, suggesting that EPS was successfully removed (Table 2). Significant difference in viability was observed with cell type (*F* = 173.0, *df* = 1,12, *p* < 0.001) (Fig. 3). Viability of EPS-hydrolyzed cells was lower than that of untreated cells, suggesting that EPS plays a role in protection during the freeze-drying process. The viability of LAB is severely affected by protective agents including EPS and soy powder. Regardless of cell types, the least viability was recorded with the absence of protective agents (*F* = 2927.5, *df* = 2,12, *p* < 0.001). With EPS as a protective agent, the viability of both cell types increased, showing 18.6% and 12.1% for untreated cells and EPS-hydrolyzed cells, respectively. The highest viability of 89.6% was obtained in untreated cells with soy powder as a protective agent, followed by 66% in EPS-hydrolyzed cells with soy powder.

Microbial EPS are extracellular carbohydrate polysaccharides that are not permanently attached to the cell surface. EPS produced by LAB has shown various health-benefit properties such as anticancer, antioxidant, antiviral, immunostimulant, and cholesterol-lowering effects [15, 16]. They also aid in the protection of *Leu. mesenteroides* WiKim32 against environmental stress such as dehydration, unfavorable temperature, osmotic stress, and antibiotics [17]. In our study, the removal of EPS resulted in low viability, indicating that EPS layer surrounding the cell surface showed protective activity against the freeze-drying process. Considering that the addition of a few EPS prior to freeze-drying improved the viability of *Leu. mesenteroides* WiKim32, moreover, EPS might be used as a protective agent.

### Effect of Cold Adaption by Supercooling Pretreatment on Stability

**Fig. 4:**
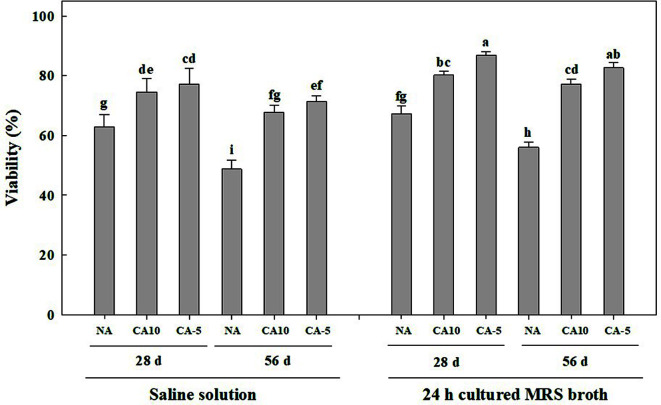
Viability of *Leuconostoc mesenteroides* WiKim32 depends on cold adaptation and the type of suspension solution used. NA: non-adaptation; CA10: cold-adapted at 10°C; CA-5: cold-adapted at −5°C.

The storage stability of freeze-dried *Leu. mesenteroides* WiKim32 cells varied with pretreatment conditions (*F* = 166.7, *df* = 2,24, *p* < 0.001), storage time (*F* = 60.4, *df* = 1,24, *p* < 0.001), and type of suspension solution (*F* = 65.6, *df* = 1,24, *p* < 0.001) (Fig. 4). Cold-adapted cells had higher viability than non-adapted cells during freeze-drying and following storage. The viability of cold-adapted cells was maintained at more than 80% after freeze-drying, while non-adapted cells had a significantly reduced viability of 67.2%. Among pretreatment methods, cold adaptation at −5°C was best for viability improvement, showing 86.8% after 28 days of storage. The viability of freeze-dried *Leu. mesenteroides* WiKim32 cells differed from the type of suspension solution including sterile saline and MRS broth cultured for 24 h. Stability of *Leu. mesenteroides* WiKim32 cells cultured in MRS broth containing 0.1% (w/v) EPS were higher than that in saline. The highest viability of 82.7% was maintained for up to 56 days in the 24-h cultured MRS broth for cold-adapted *Leu. mesenteroides* WiKim32 at −5°C, followed by cold-adapted cells at 10°C.

In our previous studies, cold adaptation has the advantage of the shelf-life extension of freeze-dried *Lactobacillus brevis* WiKim0069 [12, 13]. Supercooling condition which is below the freezing point further increased the resistance of *Lac. brevis* WiKim0069 to freeze-drying, leading to higher viability than cold adaptation at 10°C [13]. Both cation influx and SLP production increased when cells were exposed to adaptation conditions. Intracellular cations such as Ca^2+^ and Mg^2+^ can induce the activation of calcineurin, which is known as calcium-dependent serine-threonine phosphatase, and resistance to osmotic stress [18-21]. SLP formation resulting from cold adaptation made the cell surface thicker to protect cells against freeze-drying. Surface-layer thickness depended on cold adaptation conditions. Likewise, EPS plays an important role in the adherence of cells and provides a protective barrier for cells. In the presence of 0.1% (w/v) EPS, the viability of *Leu. mesenteroides* WiKim32 cells increased more than 10%. Our results are consistent with a previous study by Kim *et al*. [22], indicating that the presence of EPS produced by *Pseudoaltermonas elyakovii* improved the resistance of *Escherichia coli* against freeze-thaw cycles. The presence of EPS helps *Leu. mesenteroides* WiKim32 to resist cold stress, suggesting that it could be used as a cryoprotective agent. Cold adaptation by supercooling pretreatment would be a good strategy for the long-term storage of lactic acid bacteria such as *Leuconostoc* spp. and *Lactobacillus* spp.

## Figures and Tables

**Table 1:** Physiological analysis of non- and cold-adapted *Leuconostoc mesenteroides* WiKim32^a 6^.

Adapted condition	EPS^b^ thickness (nm)	Increase rate (%)
None	38.1 ± 3.66 c	-
10°C	42.0 ± 3.34 b	10.2
−5°C	51.6 ± 3.52 a	35.3

^a^Means ± standard deviations from three replicates. Values in the same column followed by different letters are significantly different (*p* < 0.05).

^b^EPS: exopolysaccharide

**Table 2:** Monosaccharides composition of *Leuconostoc mesenteroides* WiKim32 depends on cold adaptation and enzymatic hydrolysis^a^.

Composition (% , w/w)	Adaptation condition (°C)			Hydrolyzed cell

	None	10	−5	
Arabinose	1.6 ± 0.1 a	1.7 ± 0.0 a	1.7 ± 0.3 a	1.6 ± 0.2 a
Galactose	2.8 ± 0.1 a	2.9 ± 0.2 a	2.9 ± 0.4 a	2.7 ± 0.1 a
Glucose	8.7 ± 0.3 b	9.1 ± 0.7 ab	9.6 ± 0.3 a	5.8 ± 0.2 c
Total	13.1 ± 0.4 b	13.7 ± 0.6 ab	14.2 ± 0.5 a	10.1 ± 0.3 c

^a^Means ± standard deviations from three replicates. Values in the same row followed by different letters are significantly different (*p* < 0.05).
